# Offering memorable patient experience through creative, dynamic marketing strategy

**Published:** 2008-04-15

**Authors:** LV Purcărea, M Raţiu, T Purcărea

**Affiliations:** * ‘Carol Davila’ University of Medicine and Pharmacy, BucharestRomania; ** Romanian American University, BucharestRomania

## Abstract

Creative, dynamic strategies are the ones that identify new and better ways of uniquely offering the target customers what they want or need. A business can achieve competitive advantage if it chooses a marketing strategy that sets the business apart from anyone else. Healthcare services companies have to understand that the customer should be placed in the centre of all specific marketing operations. The brand message should reflect the focus on the patient. Healthcare products and services offered must represent exactly the solutions that customers expect. The touchpoints with the patients must be well mastered in order to convince them to accept the proposed solutions. Healthcare service providers must be capable to look beyond customer's behaviour or product and healthcare service aquisition. This will demand proactive and far–reaching changes, including focusing specifically on customer preference, quality, and technological interfaces; rewiring strategy to find new value from existing and unfamiliar sources; disintegrating and radically reassembling operational processes; and restructuring the organization to accommodate new typess of work and skill.

It is a reality that economic transformations of advanced economies are becoming dominated by services which overwhelm all other sectors. 

‘The service revolution’, that we are part of nowadays which affects all sectors including healthcare services sector is imposing new competition rules, new organization methods, new commitments about the relations and communication extension, new challenges for the marketing management. In the knowledge economy, cleverness and dynamism, innovation and technology are considered to be essential instruments . [1]

These challenges created by ‘the service revolution’ are affecting all marketing areas– including medical services marketing–which must align to these changes and must use the essential instruments (cleverness, dynamism, innovation and technology), without that, failure is certain .[2]

In order to completely satisfy healthcare services consumers' needs, it is necessary to acquire marketing abilities and to understand the patients, to identify their wishes and needs, and to build their confidence and loyalty. 

## What is a creative, dynamic strategy for the healthcare service providers? 

Defining and implementing a rock solid marketing strategy is probably the single most important factor that will contribute to the long term success of any firm, although most organizations don't have one. Only businesses that do have the vision to create a dynamic, customer–oriented marketing strategy, and also the determination to put it into practice, will achieve competitive advantage. It is these businesses that will have a real opportunity to do something special and set them apart from their competitors. 

Creative, dynamic strategies are the ones that identify new and better ways of uniquely offering target customers what they want or need. A business can achieve competitive advantage if it chooses a marketing strategy that sets the business apart from anyone else. Just doing what is typical may not give a firm any competitive advantage.

To develop a competitive strategy, healthcare companies must–first of all–*know exactly who the patients or target prospects* are and where they are located; what these people want and need, why they need it, how they need, and when they need it. The firm will have to be confident enough to develop or create a unique selling proposition that it can offer to fulfill the customers' needs and expectations. Then the proposition must be tested on the target market. The marketing manager should only attempt to implement the strategy fully when he is absolutely sure that the unique selling proposition is right.

The next task for the marketing manager is to get the message across to the market, making sure that the unique selling proposition (USP) is present in all marketing messages, campaigns, and sales channels. It is important that the marketing manager recognizes when the proposition is working so that he can then market it fast and aggressively in the sector–only this way will the firm achieve an unassailable lead over the competitors.

The business will survive in the 21^st^ century, only if the target market is chosen correctly. It is also important to focus everything the business does on providing unique value and benefits to meet the needs of that chosen market and it's necessary to do this better than its competitors. Then the business and the marketing strategy cannot stand still. The vital ingredient for a truly creative, dynamic strategy is to strive continually to discover new and better ways to add value for healthcare services to consumers .[3]

Identifying opportunities for the healthcare services company is the starting point for developing an efficient strategy. The business world is a fast–moving one and the pace of change can seem bewildering at times. The environment in which the firm operates is changing all the time and there are many different factors that influence it. There are continual changes in the service market, the customers' needs and preferences, the technology, the sales channels, and delivery methods of products or services. The most important changes and trends that affect marketing strategy planning in all domains refer to: *communication technology, role of computerization, marketing research, demographic patterns, business and organizational customers, product area, channels and logistics, sales promotion, personal selling, mass selling, pricing, international marketing*. It is obvious that most of these changes are having a positive effect on how marketers serve the healthcare services customers. These changes can bring threats to the healthcare service providers, however they will undoubtedly, bring opportunities. 

Profiling the target market is an important step for developing the right marketing strategy. Before the firm can realistically or effectively apply the right marketing strategy, it is necessary to find the answer to two vital questions: ‘what the target market is and what does the target market want or expect this business to provide?’

The first job when profiling a target market is to be able to identify precisely who the firm's audience is. The marketing manager must find out the answers to the following questions: ‘which are the characteristics of the target prospects?’; ‘which clients currently spend the most in the firm?’; ‘Why do they do this?’

The healthcare organization must define its ‘competition battlefield’, by identifying and studying its strategic segments. In this respect it is preferable the new proactive intercession, which assumes that the instrument used to satisfy a health need is conceived by the patients by means of iteration, and the quested instrument is stopping the ‘image–reaction–change’ process. The organization can open in this way, through fetched innovation, its own market and disturb the competition game to its own advantage, with a new substitution force. Applying this intercession into practice for the healthcare organization competitiveness implies compliance with some operational considerations. The most secure way to competitiveness is the one that combines in harmonic manner, productivity (the capacity to produce more with fewer tools) with effectiveness (the capacity to better respond to environment expectations, especially to the ones of the clients).[4]

A precision–driven marketing approach (that refers to a high–quality list of prospective clients) will prove far more productive and profitable to generate sales than an untargeted approach. The quality of prospects, based on the understanding, knowledge and careful profiling of the customers and their needs, will massively increase the healthcare service provider's ability to convert them into sales.

Every single time the marketing manager puts the strategy into action, he will also learn something new about the customers' needs, and whether the proposition is right for them. Marketing implementation should be a continuous process of creating a proposition to satisfy the customers, testing it and learning from it. The marketing manager can learn from it by recognizing what is right and doing more of it, or changing what is wrong as soon as he realizes it isn't working.

In order to have creative strategy, healthcare service providers should avoid some common mistakes (like *missing the target, delusion, indolence, lack of focus, lacking knowledge or awareness, groupthink, dissociation, oversimplification*) which occur over and over and can make a business fail to develop and implement strategies that create value for the customers.

## Patient satisfaction and memorable patient experience

*For the customer driven companies, clients’ satisfaction represents a marketing objective and instrument, at the same time*. As Peter Drucker remarked, three decades ago, the first objective for a company is to ‘create its own customer’. Customers will choose from the multitude of offers present on the market, the one that maximize the value in relation with the costs involved in searching the products, and with the limited mobility, knowledge and income they posess. As a consequence, customers will appreciate if the offer will reach the expected value level, which will influence the satisfaction and the probability to buy in the future.

Healthcare service companies have to understand that the customer should be placed in the centre of all specific marketing operations. The brand message should reflect the focus on the customer. The products' and services' quality should be placed at the core of the organizations ' commercial strategy. The range of products and healthcare services offered must represent exactly the solutions that customers expect. The touchpoints (or contact points) with the customers must be well mastered in order to convince them to accept the proposed solutions. Healthcare services providers must be capable to look beyond customer's behaviour or product and healthcare service aquisition.

Profit and growth come only after deeply understanding the customers, by listening to their needs and by offering what they are asking for. This is something that can be easily lost or even impossible to gain, if this is the single method used by the marketing team. For marketing people in all domains, it represents an important responsibility to share this desire to listen, to develop and to deliver at every level inside the organization . [5]

In this context, it is worth to mention a study undertaken by the Chinese University of Hong Kong which found that when consumers are uncertain about how to evaluate the efficacy of a service provided, they use external cues to make inferences about the trustworthiness of the service provider. For instance, to evaluate a doctor, they might consider how welcoming the waiting room is, the friendliness of the receptionist, or even if the room is clean and tidy. The fact that these cues may have nothing to do with the quality of the service provided does not appear to be important. A customer service desk may have caring reps in spite of its messy countertop. But first impressions count big with customers. And yes, that goes all the way down to the shiny clean floor. [6] 

Memorable customer experience leaders are focusing on a crucial element in planning their relationships with patients: they are always trying to ensure that there is no callousness on the part of hospital staff, constant availability of life–saving drugs, functioning equipment, clean bed–sheets and other linen and well–appointed patients' rooms. More than that, they are visualizing and establishing a scenario where the patient is treated as a customer and, since customer is king, he supersedes the doctors, nurses and everybody else in importance when he walks into the hospital. [7]

It is well known that strategies built on consumers' feedback have more chances of success than the ones based on managerial intuition. It's a real challenge to any marketer to find out why the customer choses a service or another. Because there is always a certain dynamic of the selection, depending on the analized market structure, on the company's position on the market (image, marketing communications) and on product and service development. 

Although modern marketing focuses on strategies (mixes) that give impulse to sales and to attracting new customers, the company's most effective defense weapon is customer retention. And the most effective approach to achieve this objective consists in offering high satisfaction to the customer, and this will lead to an improved customer fidelity.

In this context, it is worth to mention the opinion of two prestigious authors [8] who consider that customer relationship management represents the most important dimension of the company's strategy. In this respect,* Robert S. Kaplan and David P. Norton have analyzed the four essential processes in customer management: client selection, aquisition, retention and growth*. This is because the relationship has to maintain a long term contact with the customers, due to a proactive approach which strategicaly integrates the four processes – considering every process individualy – this way maximizing the client's value, and the value creation, in general. 

If the healthcare service consumer is satisfied with the provided service in relation with his expectations, he will become loyal to the healthcare provider, who applied, in this case, adequate marketing strategies (improving service quality; improving service differentiation confronted to competition services/offer, distribution, and image differentiation; increased service productivity; motivate personnel to better serve the clients). Fully satisfied customers are more likely to become loyal customers, even advocates for the healthcare service providers. Although firms put enormous amounts of money and effort into loyalty initiatives, they often are not successful in building true customer loyalty. The main ways in which firms can manage customer satisfaction and reach customer loyalty refer to: understanding what can go wrong; focusing on controllable issues; managing customer 's expectations; offering satisfaction guarantees; making it easy for the customers to complain; creating relationship programs; making customer satisfaction measurement an ongoing priority.

Measuring customer loyalty and developing a retention strategy are critical to an organization's success. The organization that understands and manages customer loyalty has a head start on its competition. It is very important to find the best strategies to build strong customer loyalty in the healthcare service organization. 

## The perfect customers's experience in the healthcare service sector

A customer experience is not just one piece of theater or a momentary delight. While those are great, and a step in the right direction, real positive customer experience comes from companies who show they care about the customer. Healthcare service providers that have a memory (so customers don't have to tell their story repeatedly) and really provide ongoing value to a customer understand that customers are truly the most valuable entity of their business. Those companies treat their customers as not only their only source of revenue, but as a scarce, valuable resource. When companies work this way we are much more likely to see really great customer experiences.

We consider that the two most important things for delivering the best experience to healthcare services customer (patient): a great product, that emotionally connects with the customers and fulfills a basic need or desire; and a deep understanding of the customer, an understanding that allows the healthcare company to anticipate what they need better and sooner than they know themselves.

To improve customer experience, companies that act in the healthcare services sector are advised to follow some key initiatives (rules) [9]:

- *Act on feedback*: Healthcare services providers that fail to respond to customer feedback are throwing away the chance to increase the number of satisfied and loyal customers. Changes need to be deployed throughout the company and communicated to employees and customers.
- *Design processes from the outside in*: Healthcare organizations need to identify which processes matter most to customers rather than designing them with the objective of improving operational efficiencies.
- *Act as one organization to ensure consistency*: Healthcare companies need to ensure that information received from a customer at one point of contact is not forgotten in the next channel.
- *Be open*: Opening channels or extending hours are one way, but it can mean more, like building communities. Healthcare organizations should be transparent and clear, open–minded and inclusive.
- *Personalize healthcare services and experiences*: Personalization can be complex, and complexity can mean costs for the healthcare company. Companies need to beware of just evaluating the costs of personalization against the sales benefits and to factor in the longer–term value of improving the customer experience.
- *Alter attitudes and healthcare company's behaviour*: Employee actions are often the most powerful actions in a customer experience. There are three ways to alter employee behavior: recruit the right employees; ensure standards with policies, procedures and governance structures; and create training programs that create incentives and can modify employee behavior.
- *Design the complete customer experience*: Healthcare organizations need to plan and design the customer experience, rather than letting it ‘just happen’.

It becomes obvious that companies that wish to be successful–but also those which want to survive – need a new way of thinking: final success belongs to those which will *put the healthcare services consumer in the centre of their activity, and will offer him a superior value*. These companies will focus on creating their own customers–not only on creating products and healthcare services–and will prove their capacity of demand creators, not only of products and services creators. 

It's very important to understand the healthcare consumer behavior, how healthcare customers buy products, what products are purchased together and what is the meaning of a satisfied consumer experience–which can be defined as the cognitions and feelings that the consumer experiences during the use of a product or service; managers' goal must be converting of merely satisfied customers into completely satisfied customers: only the completely satisfied customers should be considered loyal. 

We consider that the two most important things for delivering the best customer experience are: a great product (healthcare service), that emotionally connects with the customers and fulfills a basic need or desire; and a deep understanding of the customer, an understanding that allows the healthcare service provider to anticipate what they need better and sooner than they know themselves.

We mention that in the medical device industry, the most demanding customers keep raising the bar for service excellence. Customer requests for same–day (instead of overnight or slower) service fulfillment combined with service–level agreements that put the risks (and rewards) onto the manufacturer will be more and more common. For some companies, such as Siemens Medical Solutions, these demands typically require the creation of more complex and costly distribution and service network in order to get closer to customers and enable faster response. The enhancements needed in processes and systems for managing and optimizing these networks will challenge even the leading service businesses in coming years. [10]

## Patient experience management–a competitive strategy to improve patient centricity

What could be more important than improving sales and customer relationships? Today, there is a fast–growing movement among organizations interested in improving their customer–centricity through a better understanding of customer interactions, or touchpoints. Called ‘Customer Touchpoint Management’ (CTM), the goal of this new movement is to improve customer experiences, and as a result, improve customer relationships. By *improving customer relationships, healthcare organizations improve market share, sales, and both customer and employee loyalty and advocacy*. [11] 

A touchpoint is all of the communication, human and physical interactions that customers experience during their relationship lifecycle with the healthcare service organization. Touchpoints are important because customers form perceptions of the healthcare organization and brand based on their cumulative experiences. CTM–oriented organizations know that they can best enhance relationships with healthcare services customers by improving touchpoints across the entire organization.

**Fig 1:**
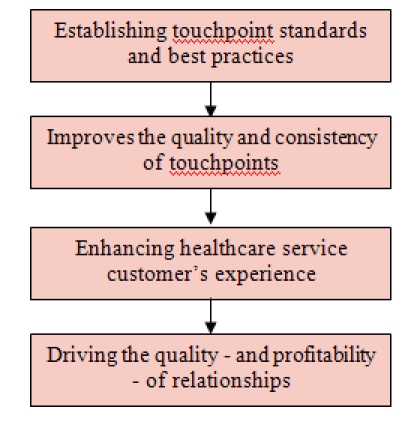
Customer Experience Management Process in the healthcare service sector

*The key to delivering outstanding customer experiences is improving the quality and consistency of touchpoints: quality in terms of meeting needs, and consistency in delivery and image*. And the key to improving the quality and consistency of touchpoints is establishing touchpoint standards and best practices.Fig 1

Setting standards establishes performance expectations. Employees need to understand what the standards are in order to perform consistently. Without standards, the quality of touchpoints is left to the individual employee. In other words, without established standards, the quality of a customer experience can be in the hands of the worst firm's employee. 

Taking into consideration these ideas, we can define Customer Experience Management as a coordinated effort to accomplish specific goals by improving the quality and consistency of customer interactions–or touchpoints. Using this strategy, a healthcare service company can gain important advantages like: constantly positive customer experiences, achieving differentiation, customer retention and referrals.

## Adequate strategies to survive the service revolution

In the last few years, with the huge changes of the global environment, the problem of surviving the services revolution [12] (global competition, automation and self–serving are combined with outsourcing and offshorring) has increased the attention given to service business–as part of services economy; beside the traditional business (financial, health care, educational, logistics and transportation, of hospitality, and so on) some other segments are consolidating as well, the so called KIBS (‘knowledge intensive business services’), such as consultancy. A common characteristic of KIBS companies is the critical role played by the customers in the service solution co–production with the service provider, which could have a deep effect on both the delivered service quality, and the client's final satisfaction related to the service solution based on knowledge.

To survive the service revolution, service companies–also the ones acting in the healthcare services sector–must start defending themselves which is about proactive, far–reaching, often draconian changes, focusing on customer preferences, quality and technological interfaces. Specifically, companies have to rewire their strategies to find new value from existing sources; disintegrate and radically assemble their operational processes; and restructure the organization to accommodate new types of work and needed skills. The driving forces behind the service transformation refer to the industrialized information chain, and the necessity of realigning the firm strategy, redesigning processes and restructuring the service organization. 

Healthcare services companies that spend time and money in understanding customer preferences and developing specific healthcare services for niche customers will do well. This becomes even more important as firms move all or some portion of their business on the Internet. 

In this context, it becomes obvious that the healthcare service company that best understands and anticipates customer's needs, and delivers consistently high quality service wins. To meet these challenges, top managers should *realign* the organization's strategies, *redesign* processes and *restructure* organization.

*Realigning strategy.* In attempting to link with customers directly, healthcare organizations must overhaul their offerings, cost structures, and competitive platforms to align with the shortened information chain and with the changing demands and behavior of their customers. Today's technology is the one that changes the relationship between sources, services, channels, and customers. And as the service becomes commoditized, competition intensifies, and differentiation becomes vital.

*Redesign processes.* As the service revolution carries on, healthcare companies have to understand their information, work processes and examine each stage of the process. In other words, processes need to be much more specific and carefully managed than ever before. They must be closely synchronized with those of other firms as well as with customers who may collaborate and participate in the producing output. Because of technological and infrastructure changes, competition, and healthcare industry restructuring are ongoing, healthcare service companies need to constantly experiment their systems if they are to negotiate these changes in a sustainable manner.

*Restructuring the organization.* Reorganization of processes necessitates organizational change. Companies' leaders must constantly redesign their healthcare organizations to adapt to new conditions, while ensuring that the customer (patient) does not get lost in the process (is completely satisfied). 

Dealing with industrialization realistically and creating an adaptive learning organization requires new skills. In order to ensure they understand the impact of new technologies, strategies, and channels on customer behaviour, healthcare companies have to include skilled managers in new areas – a chief experience healthcare service design officer, a director of experience engineering, a chief of global healthcare service delivery etc. Also, technology experts must be distributed throughout the company, rather than concentrated in a separate IT group. Then, managers of partner relationships play an important role in the new structure and they have to learn to deal with new global allies and companies. Finally, management must adapt to a more diverse workforce as the employees of the new service company may be spread across the world. Some of these necessary skills are not new to multinational healthcare service companies. But they may be very new to traditional healthcare organizations.

As a conclusion, the survivors of the service revolution will be those who really understand that opportunities lie in removing and supplanting links of the information chain and also in understanding how the chain is being restructured. Once they completely understand their own information chains, *healthcare companies must begin reorganizing strategies, processes, and people for the challenge ahead*. So, being competitive will be more and more difficult but the alternative could be a disaster.

Only a creative, dynamic healthcare services marketing strategy can help companies to survive the services revolution, this being able to offer an efficient answer to health threats, help to prevent diseases, an increased co–operation between healthcare systems to adapt to key aspects about healthcare, and to those aspects which could arise unexpectedly and require urgent attention.
